# Stone Mountain Medical Association

**Published:** 1886-07-20

**Authors:** 


### STONE MOUNTAIN MEDICAL ASSOCIATION.

This Association was permanently organized on the 19th ultimo. There were present a goodly number of intelligent medical gentlemen, representing the counties of DeKalb, Gwinnett, and Rockdale.

We are pleased to announce the establishment of this medical society as indicating a spirit of progress among the members of the profession in that section of Georgia. It were well that such societies were organized in every county in the State. Nothing so greatly promotes progress and brotherhood in the ranks of the profession as the frequent assembling of practitioners in these local societies. Such organizations are now especially appropriate in view of the fact that the Georgia Medical Association will probably, at its next meeting, adopt the Alabama system of representation from County Associations throughout the State. The Managing Editor of this journal, who though he keeps an office in Atlanta, resides in the suburban village of Decatur, the county site of DeKalb County, was present and participated in the organizatiun of the above Association, and was unexpectedly to himself elected President of the new Association. Dr. J. L. Hamilton, of Stone Mountain, was elected Vice-President, and Dr. J. H. Green, Secretary. Drs. J. M. Guess and M. L. Mahaffey, of Gwinnett, and Dr. L H. Jones, of DeKalb, were elected Censors. Previous to the election of officers, a constitution and by-laws were adopted.

After the organization a recess of one and a half hours was taken, during which time the members of the Association partook of a sumptuous repast, given by Dr. J. L. Hamilton, of Stone Msuntain. The dinner was elegantly gotten up, and served with that cordial manner and generous hospitality for which Dr. Hamilton is noted. That it was warmly appreciated and greatly enjoyed by the doctors present was plainly to be seen in the sensible relief brought to a groaning table, and in the good feeling and hilarity which marked the occasion.

In the afternoon, a short session only was held. On the call for Reports of Cases, Dr. J. M. Guess reported a case of hour-glass contraction with retained piacenta. After considerable effort, he had succeeded in removing a greater portion of the placenta, but a fragment still remained, which he had failed to remove, though some days had now elapsed. Ergot and the usual methods to produce contraction and expulsion of the offending mass were faithfully tried, and now a fever, a pulse of 120, and the putrid character of the lochial discharges give cause of anxiety. He was using veratrum to control the fever, turpentine emulsion internally, and carbolic washes to the uterus. He desired suggestions as to what should now be done. He did not think that peritoneal inflammation was, as yet, present in the case.

Dr. J. L. Hamilton stated that he had encountered only a few cases of hour-glass contraction, and of adherent placenta, in a long experience. He advised the continuance of the antiseptic washes, with turpentine stupes to the abdomen, noticing carefully the constitutional symptoms—the patient to have quinine, opiates, and antiseptics ; no harsh measures to be used, and results left to Nature.

Dr. Word stated that it was highly important the placenta be early delivered while the parts are relaxed, and the sensibility not so great. It is usually practical in such cases to introduce the hand, as soon as the difficulty is discovered, and by cautious manipulation overcome the hour-glass contraction and remove the adherent portion of placenta. If, however, this measure has been omitted, or has failed of success, it were perhaps better to leave the case to Nature, using antiseptic washes with constitutional treatment to guard against septic fever, as Dr. Guess is now doing. As antiseptic washes, weak solutions of carbolic acid, permanganate of potassium and corrosive sublimate are deemed most efficient.

Dr. Word alluded to the prevalence of diarrhœa and dysentery amongst the children at the present time, and called for suggestions ; he had nothing especially new to present.

Dr. Hamilton stated that the want of success in these cases was largely due to inattention to diet ; heat had also much to do with the persistence of these cases. He had found good success with the ordinary agents—as of chalk and mercury, bismuth, Dover's powder, etc., in connection with careful dieting, lime water with milk, etc.

Dr. Word mentioned that he had found much benefit from the use of monobromated camphor, in minute doses, triturated with sugar of milk. It could be variously combined with Dover's powder, chalk and mercury, bismuth, aromatic syrup of rhubarb, etc. In teething children, with tendency to diarrhœa, the monobromated camphor seemed to possess a prophylactic property, lessening the nervous sensibility to impressions, so that the child was made less susceptible to the irritation from teething.

Dr. Guess questioned the theory that teething was the cause of these Summer complaints, which seldom come in cold weather. As teething was a natural process, it would seem that it ought not to be charged with these troubles. Why are these diarrhœas confined to the Summer season ?

Dr. Word remarked that it was not, he believed, clearly understood how that heat tended to the production of bowel troubles. It is known that impressions made by heat upon the skin are reflected upon the liver, and in the case of children often upon the brain also. There is a recoil of blood from the surface to the internal organs ; the portal circulation is obstructed, the liver is engorged, the mucous membrane of the intestines is congested, and Nature seeks to relieve the congestion by diarrhœa and by sanguinious discharges. In tropical climates dysentery is most prevalent. Hence the usefulness of mercurials and salines in the early stages of dysentery. By salines we convert a dysentery into a diarrhœa.

Dr. J. H. Green stated that, in a recent visit to Southern Georgia, where dysentery was now very prevalent, he learned that the practitioners were using arsenic in dysentery, and found it a very efficient remedy. How it came into use, and the rationale of the treatment, he did not learn. It was given in the form of Fowler's solution, 5 drops, three times a day.

Dr. Mahaffey suggested that, if malarial causes existed, it might explain the efficacy of arsenic, which was known to be equal to quinine in malarial fevers.

After some other informal discussion, the Association adjourned to meet again on the first Thursday in October next.

